# The Journal of Balneology and Dietary

**Published:** 1891-07

**Authors:** 


The Journal of Balneology and Dietary. Published by Journal of Balneology Publishing Co., Allen H. Still, Manager, 22–26 Reade Street, New York. Price, $1.00 per annum.

We always welcome knowledge bearing on the treatment of disease without drugs. From this journal just such knowledge can be obtained, for it is a review of "physiological therapeutics, balneo-therapeutics, mineral springs, and climatology."

